# A Piezoelectric and Electromagnetic Dual Mechanism Multimodal Linear Actuator for Generating Macro- and Nanomotion

**DOI:** 10.34133/2019/8232097

**Published:** 2019-11-13

**Authors:** Xiangyu Gao, Zhanmiao Li, Jingen Wu, Xudong Xin, Xinyi Shen, Xiaoting Yuan, Jikun Yang, Zhaoqiang Chu, Shuxiang Dong

**Affiliations:** ^1^Department of Materials Science & Engineering, College of Engineering, Peking University, Beijing 100871, China; ^2^Beijing Key Laboratory for Magnetoeletric Materials and Devices, Beijing 100871, China

## Abstract

Fast actuation with nanoprecision over a large range has been a challenge in advanced intelligent manufacturing like lithography mask aligner. Traditional stacked stage method works effectively only in a local, limited range, and vibration coupling is also challenging. Here, we design a dual mechanism multimodal linear actuator (DMMLA) consisted of piezoelectric and electromagnetic costator and coslider for producing macro-, micro-, and nanomotion, respectively. A DMMLA prototype is fabricated, and each working mode is validated separately, confirming its fast motion (0~50 mm/s) in macromotion mode, micromotion (0~135 *μ*m/s) and nanomotion (minimum step: 0~2 nm) in piezoelectric step and servomotion modes. The proposed dual mechanism design and multimodal motion method pave the way for next generation high-precision actuator development.

## 1. Introduction

In the fields of precision machines, intelligent manufacturing, robotics [1–3], and other active devices [4] for moving and driving an object precisely, actuators play an irreplaceable role. Conventionally, hydraulic pressure [5–7], pneumatic pressure [8, 9], etc., can be transferred into mechanical motion. However, electric energy is more conveniently converted into motion and output force via electrostatic [6, 10], piezoelectric, electroactive [11, 12], electromagnetic induction, or magnetomotive force [13–15] coupling effects. Among them, electromagnetic and piezoelectric actuators are widely used because they are more efficient in comparison with other types of actuators. Electromagnetic actuators can produce fast motion and output force in a wide range [16, 17]. However, they are difficult to produce an effective nanostep motion. While piezoelectric stacked-ceramic servoactuators [18–20] are featured with compact size, fast response, and nanopositioning ability but only limited in tens of micrometer range [21], piezoelectric step motion actuators can also produce nanomotion, but usually, their moving speeds are limited in the range of only millimeter per second [22–24]; the frictional force coupling-based piezoelectric ultrasonic motors can work at a high velocity, but serious high-frequency wear loss between the friction tip and the coupling plate of sliders is a main problem, and their positioning resolution is also very limited [25–27]. Up to now, each actuator has itself a role but also accompanies with limitations in actuations. However, in some important precision machines like lithography mask aligner [28], long traveling distance with a high speed and fast response and also nanoresolution are required simultaneously.

To solve the dilemma of high resolution and high speed over a long traveling range, some researchers presented some novel designs with piezoelectric actuators. For example, Zhu et al. [29] presented a linear piezoelectric ultrasonic motor for producing fast motion and a piezoelectric actuator with flexible hinge for precision position. But there is still a friction problem due to high-frequency ultrasonic working mode. Other proposed solutions [30–33] include the use of stacked piezoelectric stages and electromagnetic actuators. Normally, a small precision stage (fine actuation) is simply placed on the top of a big stage (coarse actuation). For example, Jie et al. [30] reported a stacked stage in which two linear voice coil motors are used to drive a big stage on the base for producing macromotion, and one small stage driven by two high-frequency PZT actuators is mounted on the top of a big stage. Zhang et al. [31, 32] designed a similar micro-macro stacked stage with a small one mounted on the top of a macro one. Shinno et al. [33] used a laser interferometer feedback system to enable the high precision motion of one stage driven by a voice coil motor. The long working distance is obtained by using alternating current (AC) servomotor. Although resolution within a nanometer range is observed, the complexity of the close-loop control system is obvious. Note that the use of piezoelectric stacked stages or voice coil motors may obtain a nanoscale actuation, but they work effectively only in a local, limited range due to their limited displacement.

In this work, we design a piezoelectric and electromagnetic dual mechanism costator and coslider (as a common slider) and fabricate a dual mechanism multimodal linear actuator (DMMLA) prototype. Three working modes of DMMLA are proposed for producing macro-, micro-, and nanomotion via the common slider, respectively. A Finite Element Method (FEM) is adopted for the structure analysis and working principle explanation. The linear motion performances of DMMLA in different scale are also evaluated.

## 2. Structure and Operating Principle

**Figure 1:**
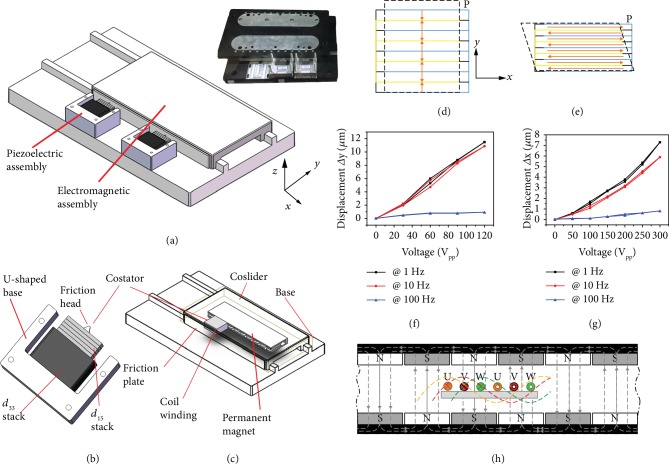
Schematic design of DMMLA and its working principles: the overall assembling of DMMLA with the prototype shown in the insert (a); the enlarged detailed structure of piezoelectric assembly (b) and electromagnetic assembly (c); the detailed structure of *d*_33_ stack (d) and *d*_15_ stack (e) and the relationship of displacement and the applied voltage at 1, 10, 100 Hz for *d*_33_ stack (f) and *d*_15_ stack (g); working principle of the electromagnetic actuator (h).

The structure design of the proposed DMMLA is shown in Figure 1(a). It consists of a dual-mechanism costator including an electromagnetic inductive coil assembly and piezoelectric stack assembly that codrive a common coslider. The dual-mechanism coslider contains one set of permanent magnets and an attached friction plate. The detailed electromagnetic assembly is presented in Figure 1(c). The piezoelectric stack assembly as shown in Figure 1(b) contains two identical piezoelectric actuators, containing a *d*_33_ stack for producing the normal motion and a *d*_15_ stack for producing the horizontal motion. Here, *d*_33_ and *d*_15_ are normal strain and shear strain piezoelectric coefficients of piezoceramics, respectively. Note that the first subscript number of *d*_*ij*_ refers to the direction of the electric field and another stands for the strain direction.

There is a zirconia friction head attached at the top of the stacks with epoxy resin (Catalyst 15 Black, Emerson and Cuming, Germantown, WI, USA), and it can transfer periodic micro/nanomotion of the piezoelectric stacks into linear motion of the coslider through friction force coupling between the friction head and the friction plate.

The detailed structure design of *d*_33_ and *d*_15_ stacks is shown in Figures 1(d) and 1(e). The *d*_33_ stack (PTJ1501414101, Suzhou Pant Piezoelectric Tech. Co. Ltd, Jiangsu, China) consists of multiple piezoelectric ceramic layers alternately polarized in thickness direction (*y* direction), while the multiple layers of *d*_15_ stack (P51, Baoding Hongsheng Co., Hebei, China) are alternately polarized along length direction (*x* direction). Figure 1(f) shows the measured *y*-direction (normal direction) displacement of *d*_33_ stack as a function of the applied voltage at different frequencies (1 Hz, 10 Hz, and 100 Hz), and Figure 1(g) shows the *x* direction (horizontal direction) displacement of *d*_15_ stack. It can be seen that the maximum normal displacement of *d*_33_ stack under 120 V_pp_ (0~+120 V) at 1 Hz and 10 Hz is more than 10 *μ*m and it decreases sharply at 100 Hz, while the horizontal displacement of *d*_15_ stack is around 6 *μ*m under 300 V_pp_ (-150 V~+150 V) at low frequencies and decreases at 100 Hz. This phenomenon can be attributed to the frequency dependence associated with the piezoelectric response of ferroelectric ceramics [18, 34].

### 2.1. Electromagnetic Linear Motion

Figure 1(h) shows the working principle of the electromagnetic linear actuation. The coil assembly at the base consists of three coils named as *U*, *V*, and *W*, which are inserted in the space between two pairs of permanent magnet sets fixed on the coslider. The three phase currents applied on the coil assembly are as follows, where *i*, *I*, *ω*, and *t* stand for current through each coil, effective value of the input current, circular frequency of input signal, and time.

(1) $$\begin{array}{c} i_{U}=\sqrt{2}I\cos\omega t,\\ i_{V}=\sqrt{2}I\cos\left(\omega t-120^{{}^{\circ}}\right),\\ i_{W}=\sqrt{2}I\cos\left(\omega t-240^{{}^{\circ}}\right). \end{array}$$

Consequently, a sine-shaped traveling wave magnetic field will be excited along the longitudinal direction of the coslider. Because of electromagnetic induction between the coil assembly and the permanent magnet sets, the magnet sets together with slider will be driven to move linearly (i.e., macromotion) along the longitudinal axis direction. The resultant magnetomotive force *f* from the coil assembly can be estimated by the following equation [35].

(2) $$\begin{array}{c} f\left(\theta_{s},t\right)=F\cos\left(\omega t-\theta_{s}\right), \end{array}$$

where *F* is the amplitude of magnetomotive force; *θ*_*s*_ is the space position angle. Certainly, *f* is also the output force of the coslider.

The magnetic force *f* can be calculated by the following equation [35].

(3) $$\begin{array}{c} f=B_{\delta }lNI_{a}, \end{array}$$

where *B*_*δ*_, *l*, *N*, and *I*_*a*_ are the magnetic induction intensity, effective length of the coil in the magnetic field, turns per coil, and current through the coil, respectively. The maximum transient state electromagnetic force can reach to 88 N (corresponding to a peak current of 6.0 A) and the continuous pushing force is only 9.1 N (corresponding to a continuous current of 2.6 A).

### 2.2. Piezoelectric Step Motion

**Figure 2:**
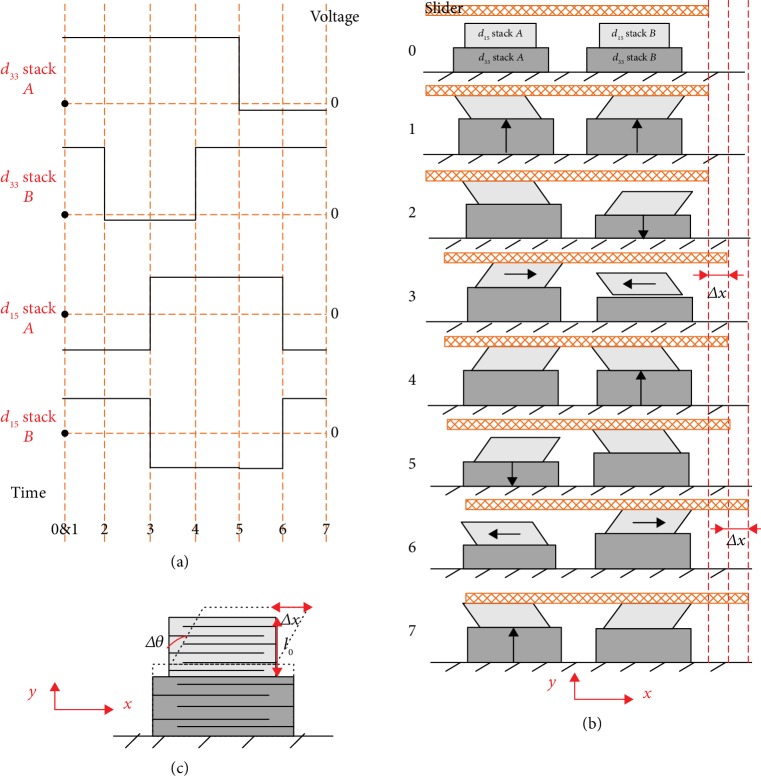
Working principle of piezoelectric step motion. (a) Sequence voltage signals applied to the piezoelectric stacks *A* and *B*; (b) driving process and step motion of the coslider; (c) step displacement Δ*x* produced by the piezoelectric stack.

When the power offering to coil assembly is switched off, the coslider is then driven by piezoelectric assembly, i.e., two hybrid stacks *A* and *B* fixed at the U-shaped base. The stacks *A* and *B* are *d*_33_ and *d*_15_ stacks, respectively, which would produce displacement in the +*y* and +*x* direction when positive voltages are applied and vice versa. Therefore, the stacks *A* and *B* can produce two orthogonal quasistatic state displacements in *y* and *x* direction, respectively, and drive the coslider moving linearly along *x* direction in step motion through frictional coupling between the friction head and the friction plate.  Figure 2(a) shows a set of the driving voltage waves, and Figure 2(b) shows a cycle of microstep motion of the coslider in six operation sequences.

1. Initial state: both *d*_33_ stacks *A* and *B* and *d*_15_ stacks *A* and *B* are at initial state without strain because of zero electric potential (*V*_33,*A*_ = *V*_33,*B*_ = 0; *V*_15,*A*_ = *V*_15,*B*_ = 0)
2. Both *d*_33_ stacks *A* and *B* are at an elongated state in *y* direction, while *d*_15_ stacks *A* and *B* are in a reverse shear state in *x* direction because of the high electric potential (*V*_33,*A*_ = *V*_33,*B*_ = *U*_33_; *V*_15,*A*_ = −*U*_15_, *V*_15,*B*_ = *U*_15_)
3. The *d*_33_ stack *A* keeps its elongated state to hold the coslider, while the *d*_33_ stack *B* is at its shorten state to be off the coslider by applying a small negative potential
4. The *d*_15_ stacks *A* and *B* produce opposite shear displacement in *x* direction; however, the *d*_15_ stack *A* keeps touching the coslider while the stack *B* is off; therefore, the slide is driven to move one step Δ*x* in +*x* direction
5. The *d*_33_ stack *B* elongates to touch with the coslider, while the *d*_15_ stacks *A* and *B* keep in a reverse shear state
6. The *d*_33_ stack *A* shortens to be off the coslider, while the stack *B* keeps touching with the coslider
7. The *d*_15_ stacks *A* and *B* produce opposite shear displacement in *x* direction; however, *d*_15_ stack *B* keeps touching the coslider while the stack *A* is off; therefore, the slide is again driven to move one more step Δ*x* in +*x* direction.

After one cycle of step motion, the slide moves two steps (2Δ*x*) in *x* direction. The reversed step motion of the coslider in −*x* direction can be obtained by exchanging signal voltages applied to *d*_15_ stacks *A* and *B*. In Figure 2(c), the displacement Δ*x* of each step can be calculated as follows:

(4) $$\begin{array}{c} \Delta x=N_{15}d_{15}U_{15}, \end{array}$$

where *N*_15_, *d*_15_, and *U*_15_ are the number of *d*_15_ stack layers, shear piezoelectric constant, and the applied voltage to *d*_15_ stack, respectively.

### 2.3. Piezoelectric Servomotion

**Figure 3:**
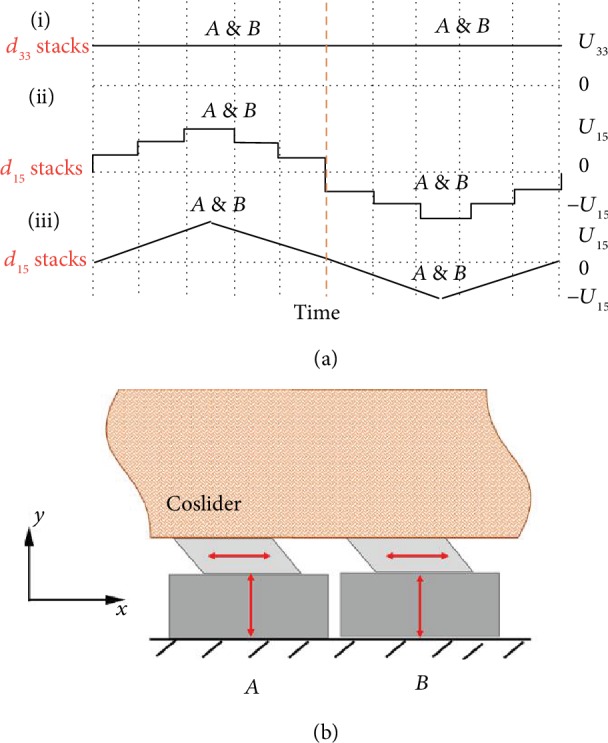
Working modes of piezoelectric servomotions. (a) Sequence voltage waves for driving piezoelectric stack assembly (*A* and *B*); (b) two piezoelectric stacks (*A* and *B*) synchronistically or alternately drive the coslider.

In this work, we designed the following motion mode for producing nanoservomotions. The two stacks *A* and *B* synchronistically or alternately work in nanostep servomotion, as shown in Figures 3(a-i, a-ii) and 3(b), or nanolinear servomotion, as shown in Figures 3(a-i, a-iii) and 3(b). Because two *d*_33_ stacks *A* and *B* synchronistically or alternately elongate to touch the coslider, two *d*_15_ stacks *A* and *B* also synchronistically or alternately drive the contacted slide moving step by step or linearly due to the static friction force. Consequently, the slider would move in four servomodes as follows:

1. Local step servomotion: two stacks *A* and *B* synchronistically drive the coslider moving in nanostep in a given range but with a double output force
2. Continual step servomotion: two stacks *A* and *B* alternately drive the coslider moving in nanostep in a whole traveling range but with a half load ability
3. Local linear servomotion: two stacks *A* and *B* synchronistically drive the coslider moving linearly in a given range but with a double output force
4. Continual linear servomotion: two stacks *A* and *B* alternately drive the coslider moving linearly in a whole traveling range but with a half load ability

The servoresponse time is fast, and mechanical load is also higher because the two piezoelectric hybrid piezoelectric stacks can generate large driving force. Moreover, the most important character of the dual mechanism multimodal linear actuator is its function of continual servoactuation with nanopositioning resolution within a whole traveling range, while traditional nanoservoactuation based on stacked stage design could work only in a limited, local range (normally in 10-100 micrometer range).

## 3. Simulation and Analysis

The simulation on piezoelectric actuations is established with the Finite Element Method via Comsol software (COMSOL Multiphysics, COMSOL Inc., Stockholm, Sweden). The piezoelectric stacks are made of commercial PZT-5H piezoelectric ceramic. The *d*_33_ and *d*_15_ stacks are in the sizes of 14 mm × 14 mm × 10 mm and 13 mm × 8 mm × 5 mm, respectively. The bottom surface is fixed. By applying DC voltages of 150 V and 120 V to the *d*_15_ and *d*_33_ stacks, we can get the output displacements of 2.8 *μ*m in *x* direction and 9.6 *μ*m in *y* direction, respectively.

**Figure 4:**
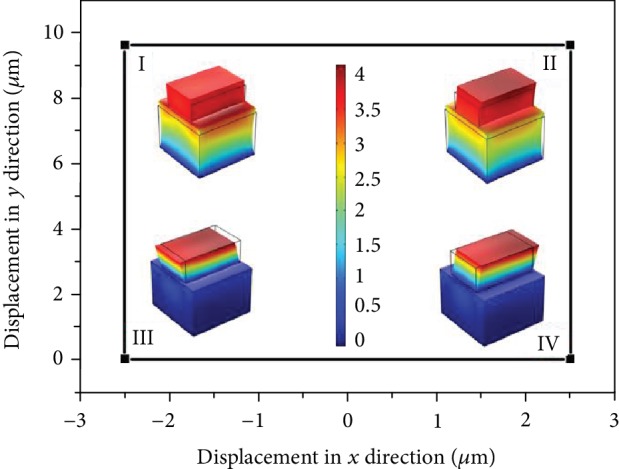
Simulated motion trajectory at the top of the stack assembly. The inserts show simulated deformation of the hybrid piezoelectric stacks at each state (unit: *μ*m).

Figure 4 shows the simulation results of the piezoelectric step motion and servomotion modes. The motion track at the central point of the top surface of the stack assembly in step motion mode is a rectangular loop with the motion sequence of I, II, III, and IV while the tip track is a line (I, II) in servomotion mode. The inserts in Figure 4 present the deformation shape of the piezoelectric stack assembly under DC voltage drive. It works as predicted in the working principle part. The detailed simulation for motion sequence in different working modes is shown in [Supplementary-material supplementary-material-1] (piezoelectric step motion) and [Supplementary-material supplementary-material-1] (piezoelectric servomotion).

## 4. Actuation Performances of DMMLA

The prototype of the proposed DMMLA is fabricated to verify the working principle and examine its macro-, micro-, and nanoactuation performances in an open loop. A coil assembly as an electromagnetic part of the dual mechanism costator is mounted on the base while two piezoelectric stack assemblies as the piezoelectric part of the dual mechanism costator are also mounted on the base. The friction tips of the piezoelectric stack assembly are pressed against a movable slider under a loading force of 20 N. The slider is designed as a common coslider containing the attached frictional coupling plate and a set of permanent magnets, to receive piezoelectric driving force from piezoelectric stack assembly and/or magnetomotive force from the electromagnetic coil assembly, respectively or synchronously.

### 4.1. Electromagnetic Motion

A linear synchronous electromagnetic actuator (Heidstar Co., Xiamen, China) is employed to build the dual mechanism multimodal actuator, in which the three-phase coils (the primary-side) are fixed at the base as an electromagnetic part of the dual mechanism costator, while a set of permanent magnets (the secondary-side) attached at the coslider is used as common coslider. When three-phase AC currents with a phase difference of 120° between them go through the primary-side coil, a traveling wave magnetic field will be excited, pushing the secondary-side permanent magnets together with the coslider to move linearly.

The magnetomotive force induced linear motion velocity is controlled by a motion control card (Galil Motion Control Company, CA, USA). The DMMLA prototype is installed on the vibration damping platform and tested at different setting speed with the help of a displacement sensor (LK-G30, Keyence, Japan).

**Figure 5:**
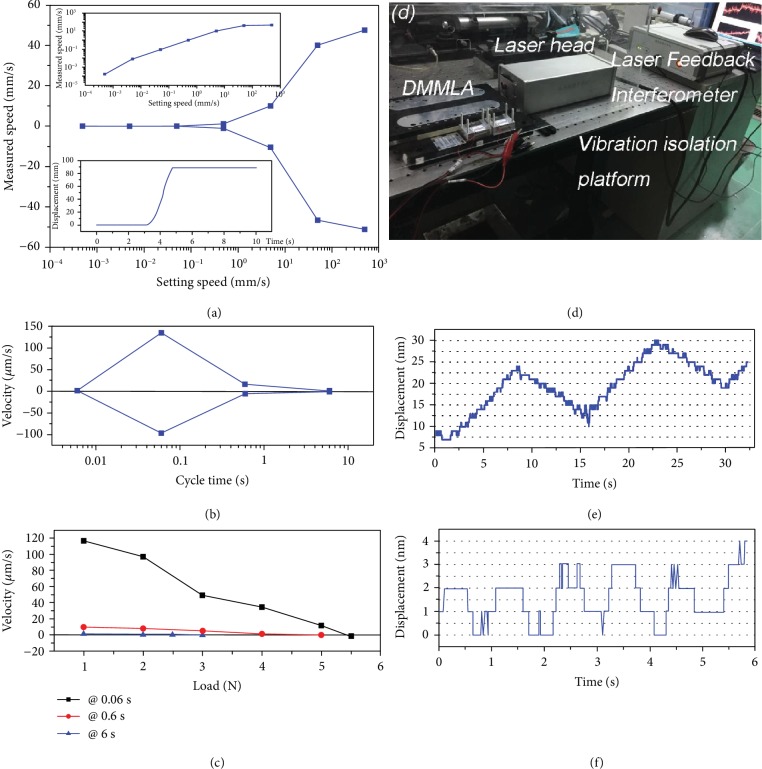
Actuation performances of DMMLA with three working modes. Electromagnetic motion: (a) measured speed with different setting speed while the insert figure (i) shows in the log format and the insert figure (ii) shows the relationship of displacement vs. time. Piezoelectric step motion: (b) velocity under the driving voltage at different frequency; (c) velocity under different load and frequency. Piezoelectric servomotion: (d) test setup; (e) step voltage mode; (f) piezoelectric servomode under rectangular voltage, and the repeatable minimum step motion is as low as 0-2 nm. Note that the shifting is caused by circumstance vibration noise.

As shown in Figure 5(a), the DMMLA exhibits a near linear speed in the speed range of 5 × 10^−4^~50 mm/s in the test. The maximum linear motion speed is 51.2 mm/s. In addition, it also shows a good symmetrical linear motion performance in +/-*x* direction in the speed range of 5 × 10^−4^~50 mm/s. The relationship between the displacement and the time is shown in the insert (ii) in Figure 5(a), and the instantly measured speed can reach to 100 mm/s.

### 4.2. Piezoelectric Step Motion

According to the driving voltage waveform shown in Figure 2(a), the two piezoelectric stack assemblies alternately work to produce the desired step motion. The velocity test is the same as the electromagnetic test, and the mechanical loading test was achieved by the weight pulling method.

Figure 5(b) shows the measured step motion velocity of DMMLA as a function of cycle time *T*. Since there are six time interval in each cycle, we chose 0.006 s, 0.06 s, 0.6 s, and 6 s as the test cycle time. It is found that as the working cycle time decreases, the motion velocity of the DMMLA increases till *T* = 0.06 s. It is because the stack assemblies produce more steps in one cycle. The maximum velocity at 0.06 s cycle time under 100 V_pp_ applied to the *d*_33_ stack and 250 V_pp_ to the *d*_15_ stack, respectively, is 134.6 *μ*m/s. At the cycle time of 0.006 s, the coslider stops moving because of the decreased displacement of *d*_33_ piezoelectric stacks at that frequency which coincides with the performance of *d*_33_ stack at 100 Hz as shown in Figure 1(f). The detailed relationship of displacement and time is shown in [Supplementary-material supplementary-material-1].

Furthermore, we tested the loading performance of the step motion mode. Figure 5(c) shows the measured step motion velocity as a function of mechanical load (weight) that is applied to the coslider axially via a line. It can be seen that the step motion velocity decreases almost linearly with the load, and it is found that the max load force of the DMMLA in step mode is 5.5 N. The relationship of displacement and time under different loading condition is presented in [Supplementary-material supplementary-material-1]. Compared with ultrasonic motors, the step mode of the DMMLA apparently shows a better loading performance due to its quasistatic motion [36, 37].

### 4.3. Piezoelectric Servomotion

According to the driving voltage waveform shown in Figure 3(a), we further measure the piezoelectric servomotion. During measurement, a Laser Feedback Interferometer (Beijing Leice Technology Co. Ltd, Beijing, China) is used to monitor the minimum servostep motion. The test setup including a vibration damping platform is shown in Figure 5(d). In order to further reduce the environmental noise, the DMMLA was placed within a plexiglass box during measurement.

The piezoelectric servomotion is produced by applying the step voltage waveform as shown in Figure 3(a-i, a-ii). In measurements, one voltage cycle including eight step-up (in +*x* direction) and eight step-down (backward) voltages lasting time for 1 second for each step is applied to the piezoelectric stacks *A* and *B* assembly. The servomotion responding to the step voltage is shown in Figure 5(e). It is clearly seen that the minimum servomotion response, i.e., step resolution, to a step voltage of 3 V_pp_ is as low as 2 nm, as shown in Figure 5(f). A drifting phenomenon was also observed, which could be explained by the environmental vibration noise. In the strict sense, the minimum step could be distinguished even it was smaller than 2 nm, as long as circumstance vibration noise is restricted effectively.

## 5. Discussion

To realize the multimodal linear macro-, micro-, and nanomotions, we think that the following working modes can be adopted. (i) In long travel range (macroscale, i.e., millimeter and above), electromagnetic motion mode will be started for producing macroscale motion. The electromagnetic motion has features of high speed and near zero friction, suiting for long range motion; (ii) in the microscale range (submillimeter and below), piezoelectric step motion mode will be started for producing high precision motion with quick response time; (iii) in the nanoscale positioning (nanometer to micrometers), piezoelectric servomotion mode will be started through the shear deformation of two piezoelectric stacks.

The three motion modes could be alternatively started for producing macro-, micro-, and nanomotions with a high speed and high position resolution. Moreover, two piezoelectric stacks *A* and *B* have a locking-position function when they are in an elongation state via *d*_33_ stacks. Clearly, the dual mechanism multimodal driving method is a unique design.

The motion performance of the DMMLA prototype and its comparison with previous work are summarized in Table 1. In minimum step resolution measurement, our system is operated in an open loop, but its resolution is even higher in comparison with the complicated close-loop stage [38]. Theoretically speaking, the displacement resolution of the proposed DMMLA can be further improved by using the similar feedback method. In addition, the design of coslider (i.e., only one common stage) can decrease the stage mass (therefore quicker starting speed) and also avoid the mutual perturbation problem that occurred in stacked stages.

## 6. Conclusion

In summary, a DMMLA containing piezoelectric and electromagnetic costator and common coslider is proposed for multiscale precision actuation. A multimodal motion method including magnetomotive force induced linear macromotion, the reverse piezoelectric effect coupled microstep motion and nanoservo motion, is presented. The working principle of DMMLA is theoretically validated by FEM simulation, and each motion mode is then experimentally confirmed. Measured results show that the maximum traveling velocity of the DMMLA is 51.2 mm/s in macromotion mode and minimum step resolution is 2 nm in nanoservo motion mode. This work shows that the DMMLA has a potential for precision nanoactuation in a long travel range, which is important in advanced intelligent manufacturing like lithography mask aligner. The proposed multimechanism and multimodal design suggests an effective method for next generation precision actuator development.

## Figures and Tables

**Table 1:** Comparison of open-loop motion performances for high speed and high resolution.

Parameters	Our work		Previous work			
	Electromagnetic motion	Piezoelectric step motion	Traditional electromagnetic linear actuator^a^	Traditional piezoelectric ultrasonic motor [25, 26, 39]	Traditional piezoelectric step motor [22]	Stacked stage [30, 38]
Max speed (mm/s)	51.2	0.134	50	1527	6	10
Traveling range (mm)	100		150	—	—	52
Displacement resolution (nm)	2		50	100~1000	5	5

^a^HDS-ULS-X, Heidstar Co., Xiamen, China.

## Supplementary Materials

Supplementary Materials SM1: simulation details. Figure S1: meshed hybrid piezoelectric stack assembly with dimensions (unit: mm). Figure S2: simulated motion sequence of the stack assembly in the mode of piezoelectric step motion (unit: *μ*m). The step number is corresponding to the six operation sequences (0)~(6) mentioned in Section 2.2. Figure S3: simulated motion sequence of the stack assembly in the mode of piezoelectric servomotion (unit: *μ*m). The *d*_33_ stacks stay elongated and the *d*_15_ stacks produce shear motion step by step or lineally along −*x*-axis direction (1), return to initial position (2), shear motion along +*x* direction (3), and return to initial position (4), finishing a cycle. Figure S4: piezoelectric step motion test without loads. (a) Piezoelectric step motion without a load at different cycle time (0.006 s, 0.06 s, 0.6 s, and 6 s). (b) Enlarged figure for displacement vs. time at the optimized cycle time (0.06 s). Figure S5: piezoelectric step motion test with different loads. Piezoelectric step motion under different loading conditions with the cycle time of (a) 0.06 s, (b) 0.6 s, and (c) 6 s.
