# Supplementary material for: A Piezoelectric and Electromagnetic Dual Mechanism Multimodal Linear Actuator for Generating Macro- and Nanomotion
Source: Research (Wash D C). 2019 Nov 13;2019:8232097. doi: 10.34133/2019/8232097 (PMC6946261; doi:10.34133/2019/8232097)
Supplement: Supplementary Materials — SM1: simulation details. Figure S1: meshed hybrid piezoelectric stack assembly with dimensions (unit: mm). Figure S2: simulated motion sequence of the stack assembly in the mode of piezoelectric step motion (unit: μm). The step number is corresponding to the six operation sequences (0)~(6) mentioned in Section 2.2. Figure S3: simulated motion sequence of the stack assembly in the mode of piezoelectric servomotion (unit: μm). The d33 stacks stay elongated and the d15 stacks produce shear motion step by step or lineally along −x-axis direction (1), return to initial position (2), shear motion along +x direction (3), and return to initial position (4), finishing a cycle. Figure S4: piezoelectric step motion test without loads. (a) Piezoelectric step motion without a load at different cycle time (0.006 s, 0.06 s, 0.6 s, and 6 s). (b) Enlarged figure for displacement vs. time at the optimized cycle time (0.06 s). Figure S5: piezoelectric step motion test with different loads. Piezoelectric step motion under different loading conditions with the cycle time of (a) 0.06 s, (b) 0.6 s, and (c) 6 s. [file 8232097.f1.docx]

Supplementary Materials

**SM1. Simulation details**

Comsol 5.4 was used as FEM tool to simulate the working principle of piezoelectric and electromagnetic dual mechanism multimodal linear actuator. The Module of Solid Mechanics (solid), Electrostatics (es) and Multiphysics-Piezoelectric Effect (pze1) were adopted. The structure and size of a single hybrid piezoelectric stack with mesh are shown in Figure S1. The bottom of the *d_33_* stack is fixed (under the constraint state). The *d_33_* and *d_15_* stacks are polarized in y and x directions, respectively. PZT-5H ceramic in the Comsol material library is used and the primary properties are as follows: density *ρ*=7.5 g/cm3, relative permittivity *ε_33_*=3400, piezoelectric constants *d_33_*=593 pC/N, *d_15_*=741 pC/N, elastic constants *c_11_*=1.27*10^11^ Pa, *c_12_*=8.02*10^10^ Pa.


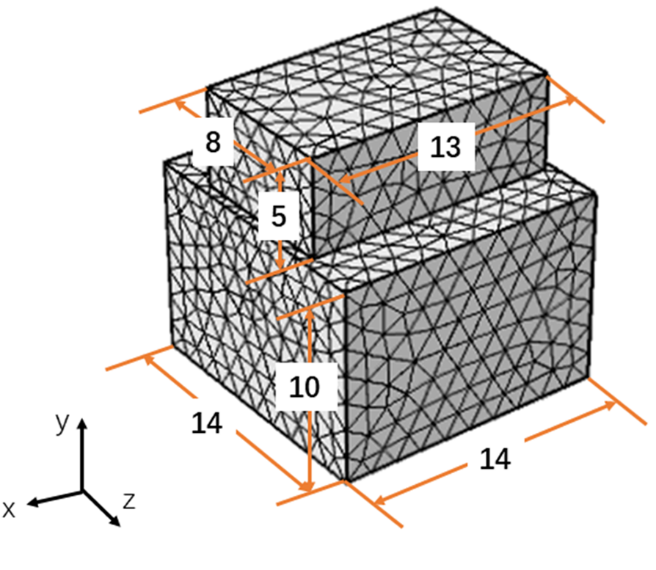


Figure S1. Meshed hybrid piezoelectric stack assembly with dimensions. (unit: mm)


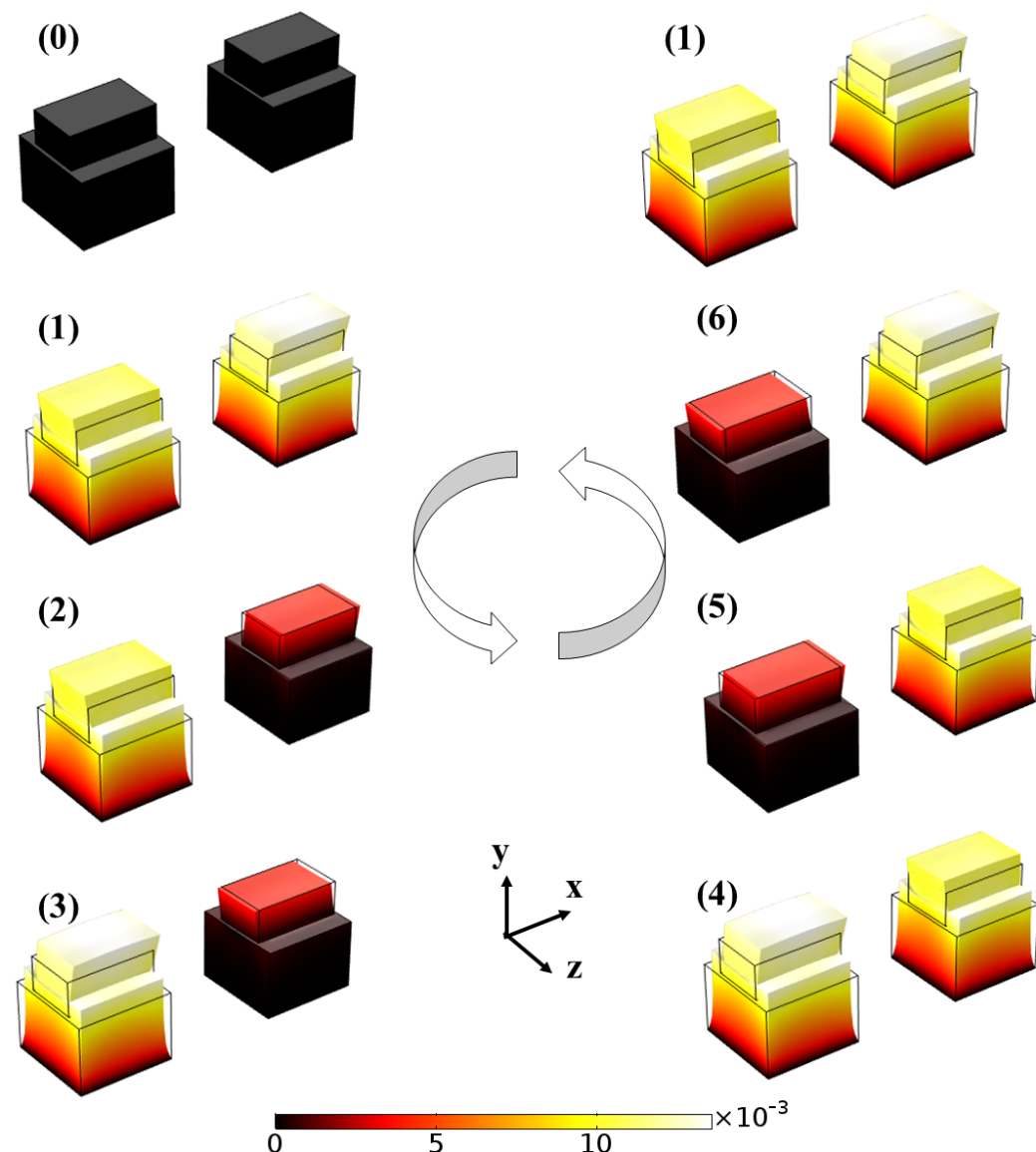


**Figure S2. Simulated motion sequence of the stack assembly in the mode of piezoelectric step motion.** (unit: μm) The step number is corresponding to the six operation sequences (0)~(6) mentioned in Section 2.2.


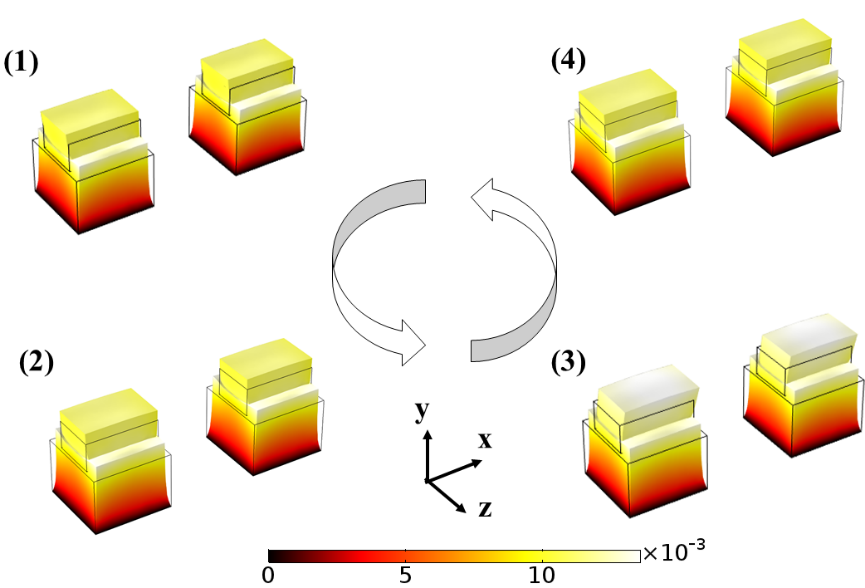


**Figure S3.** Simulated motion sequence of the stack assembly in the mode of piezoelectric servo motion. (unit: μm) The *d_33_* stacks stay elongated and *d_15_* stacks produce shear motion step by step or lineally along *–x*-axis direction (1), return to initial position (2), shear motion along *+x* direction (3), and return to initial position (4), finishing a cycle.


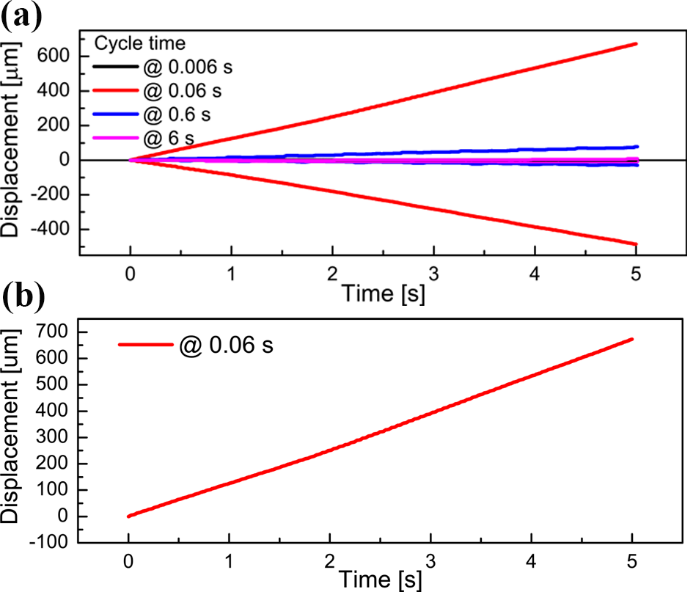


**Figure S4. Piezoelectric step motion test without loads.** (a) Piezoelectric step motion without a load at different cycle time (0.006 s, 0.06 s, 0.6 s, 6 s). (b) Enlarged figure for displacement vs. time at the optimized cycle time (0.06 s).


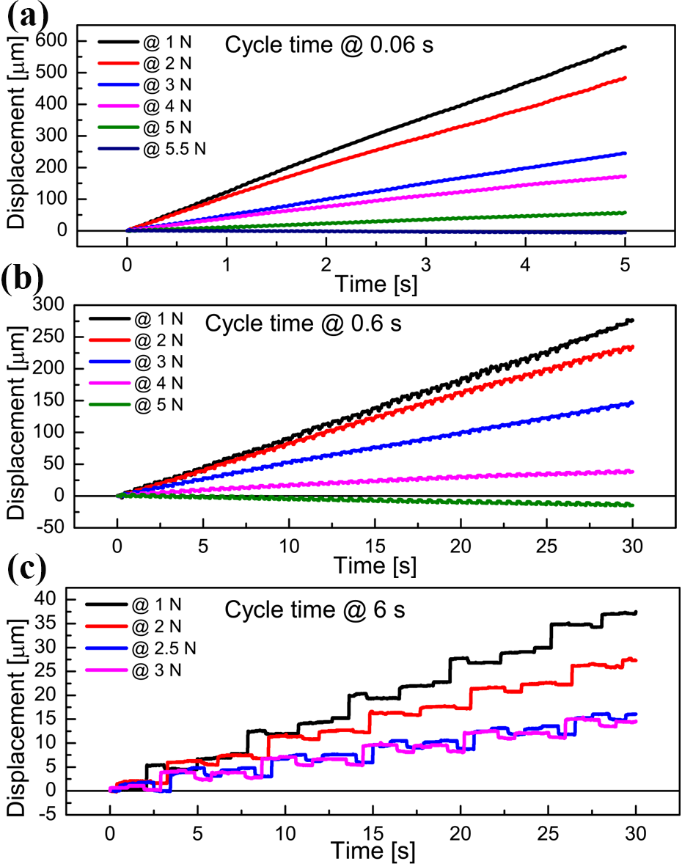


**Figure S5. Piezoelectric step motion test with different loads.** Piezoelectric step motion under different loading conditions with the cycle time of (a) 0.06 s, (b) 0.6 s and (c) 6 s.
